# Efficacy of a Technology-Enhanced Community Health Nursing Intervention vs Standard of Care for Female Adolescents and Young Adults With Pelvic Inflammatory Disease: A Randomized Clinical Trial

**DOI:** 10.1001/jamanetworkopen.2019.8652

**Published:** 2019-08-07

**Authors:** Maria Trent, Jamie Perin, Charlotte A. Gaydos, Jennifer Anders, Shang-en Chung, Lisa Tabacco Saeed, Julia Rowell, Steven Huettner, Richard Rothman, Arlene Butz

**Affiliations:** 1 Section on Adolescent/Young Adult Medicine, Department of Pediatrics, Johns Hopkins University School of Medicine, Baltimore, Maryland; 2 Department of Pediatrics, Johns Hopkins University School of Medicine, Baltimore, Maryland; 3 Johns Hopkins International STD Laboratory, Department of Medicine, Johns Hopkins University School of Medicine, Baltimore, Maryland; 4 Division of Pediatric Emergency Medicine, Department of Pediatrics, Johns Hopkins University School of Medicine, Baltimore, Maryland; 5 Department of Adult Emergency Medicine, Johns Hopkins University School of Medicine, Baltimore, Maryland; 6 Division of General Pediatrics and Adolescent Medicine, Department of Pediatrics, Johns Hopkins University School of Medicine, Baltimore, Maryland

## Abstract

**Importance:**

Pelvic inflammatory disease (PID) is a common reproductive health disorder that disproportionately affects female adolescents and young adults. Despite data indicating poor adherence and adverse outcomes among those who experience subsequent *Neisseria gonorrhoeae* and *Chlamydia trachomatis* infection, few trials have been designed to address this public health need.

**Objective:**

To examine the efficacy of a technology-enhanced community health nursing (TECH-N) intervention vs standard of care for improving PID self-management behaviors and 90-day longitudinal prevalence of *N gonorrhoeae* and *C trachomatis* infection.

**Design, Setting, and Participants:**

This randomized clinical trial of the TECH-N intervention was conducted among female patients 13 to 25 years of age diagnosed with mild to moderate PID who were being discharged to outpatient treatment from September 6, 2012, to December 8, 2016, at a large academic medical center. The final analysis of data was completed in November 2018. This study compared the efficacy of the intervention with that of the standard of care using an intention-to-treat analysis.

**Interventions:**

Enrolled participants completed an audio computer-assisted self-interview, provided specimens for *N gonorrhoeae* and *C trachomatis* testing, and were randomized to standard treatment (n = 137) or the TECH-N intervention (n = 149). Intervention participants received text-messaging support and a community health nurse visit within 5 days of diagnosis. Change in the prevalence of *N gonorrhoeae* and *C trachomatis* infection was estimated with logistic regression. The *N gonorrhoeae* and *C trachomatis* positivity rate over time was evaluated using generalized estimating equations.

**Main Outcomes and Measures:**

The primary outcome was the prevalence of *N gonorrhoeae* and *C trachomatis* infection at 90-day follow-up. The secondary outcome was adherence to the Centers for Disease Control and Prevention recommendations for self-care.

**Results:**

A total of 286 patients (mean [SD] age, 18.8 [2.5] years; 268 [93.7%] African American) participated in the study. Although the study groups were demographically similar, the intervention group had a higher baseline rate of *C trachomatis* infection (45 of 139 [32.4%] vs 25 of 132 [18.9%], *P* = .01). Although *N gonorrhoeae* and *C trachomatis* positivity was not statistically different between groups at 90-day follow-up (6 of 135 [4.4%] vs 13 of 125 [10.4%], *P* = .07), the differential rate of decrease was significantly higher in the intervention group (48 of 140 [34.4%] to 6 of 135 [4.4%] compared with 34 of 133 [25.6%] to 13 of 112 [10.4%], *P* = .02). Intervention participants were more likely to receive the Centers for Disease Control and Prevention–recommended short-term follow-up visit compared with the control group (131 of 139 [94.2%] vs 20 of 123 [16.3%], *P* < .001).

**Conclusions and Relevance:**

Adolescent and young adults with PID in the TECH-N intervention were more likely to experience decreases in *N gonorrhoeae* and *C trachomatis* positivity compared with the control group and to receive short-term clinical assessment. These findings suggest that the TECH-N intervention should be considered as a potential enhancement of standard of care approaches for management of female adolescents and young adults with mild to moderate PID in urban communities facing significant sexually transmitted infection disparities.

**Trial Registration:**

ClinicalTrials.gov identifier: NCT01640379

## Key Points

**Question:**

Is a technology-enhanced community health nursing intervention more efficacious than the standard of care for reducing recurrent and incident sexually transmitted infection with *Neisseria gonorrhoeae* and *Chlamydia trachomatis* and does it improve patient adherence to key self-care behaviors for outpatient management of mild to moderate pelvic inflammatory disease?

**Findings:**

In this randomized clinical trial of 286 female patients with mild to moderate pelvic inflammatory disease, patients in the intervention group experienced decreases in *N gonorrhoeae* and *C trachomatis* infection over time and were significantly more likely to receive short-term follow-up care according to Centers for Disease Control and Prevention standards compared with controls.

**Meaning:**

The technology-enhanced community health nursing intervention is an efficacious strategy for achieving decreases in *N gonorrhoeae* and *C trachomatis* infection during the 3 months after diagnosis and delivering short-term clinical follow-up after pelvic inflammatory disease.

## Teaser

This randomized clinical trial examines the efficacy of a technology-enhanced community health nursing intervention for improving pelvic inflammatory disease (PID) self-management and longitudinal prevalence of *Neisseria gonorrhoeae* and *Chlamydia trachomatis*.

## Introduction

Pelvic inflammatory disease (PID) is a common reproductive health problem associated with significant age and racial/ethnic health disparities.^1,2^ Potential sequelae of PID include ectopic pregnancy, tubal infertility, and chronic pelvic pain (CPP).^3,4^ The risk of fertility impairment increases with each subsequent episode of sexually transmitted infection (STI) and/or PID^5^; thus, a national health objective was created to reduce infection among young women with 2 common STIs and key causative agents for PID: *Neisseria gonorrhoeae* and *Chlamydia trachomatis*.^6^ Research with urban adolescents and young adults has demonstrated the importance of fertility and the value of preventing the sequelae associated with PID.^7^ Although researchers have focused on childbearing, the PID Evaluation and Clinical Health (PEACH) study revealed that CPP affected more than 40% of patients with PID. Given the effects on health-related quality of life, prevention of CPP is critically important.^8^

During the past 2 decades, care of adolescent and young adult patients with PID has shifted from inpatient to outpatient settings based on the data from the PEACH study and subsequent health economic analyses using these data.^9,10^ Our analysis^8^ of data from 831 women in the PEACH study indicated that the overall outcomes for patients in the trial were suboptimal. Although 52% of enrolled participants experienced a live birth, a primary outcome assessment of well-being used by reproductive health researchers, 21% of participants experienced a recurrent STI or PID diagnosis, 40% of patients experienced CPP, and 21% of affected patients experienced infertility. Furthermore, among adolescents 19 years or younger in the trial, those who experienced recurrent disease were 5 times more likely to report CPP.^8^ Although research has demonstrated the significant cost differential between inpatient and outpatient treatment for PID, health economic analyses indicate that hospitalization of young women is no longer a cost-effective approach to management of mild to moderate PID.^11^ Adherence and longitudinal clinical outcomes from randomized trial data^8,9^ suggest that some additional intervention is merited.

Furthermore, research has demonstrated poor practitioner adherence to the Centers for Disease Control and Prevention (CDC) sexually transmitted disease (STD) treatment guidelines across the United States and poor patient adherence in randomized clinical trials^12^ and in institutional care settings with standardized approaches to outpatient PID management.^13^ The CDC recommendation for a 72-hour follow-up visit suggests that interactive intervention is necessary to reduce the disparities observed among the mostly young women affected by this disorder, although few young women follow up for care.^14^ Alternate clinical and behavioral interventions designed to improve patient adherence are essential to ensure recovery and for prevention of recurrent disease, CPP, and infertility among affected patients. The Technology Enhanced Community Health Nursing (TECH-N) study examined the first intervention to date, to our knowledge, designed to meet this specific need in adolescent and young adult care.

The objectives of this analysis were to compare the effectiveness of the TECH-N intervention vs standard of care for improving (1) short-term self-management adherence and (2) the 90-day longitudinal *N gonorrhoeae* and *C trachomatis* outcomes for female adolescents and young adults enrolled in the TECH-N study. Although we evaluated patient adherence to self-care behaviors, we hypothesized that the TECH-N group would have a better rate of short-term (5-day) clinical follow-up compared with the control group and a lower rate of recurrent STIs at 90 days compared with the control group.

## Methods

The design of this randomized clinical trial has been previously reported in the literature^14^ but is described briefly here (trial protocol is available in Supplement 1). Patients with mild to moderate PID aged 13 to 25 years were recruited from pediatric and adolescent medicine clinics and pediatric and adult emergency departments within a large urban academic medical center located in a community with high STI prevalence from September 6, 2012, to December 8, 2016.^15^ The final analysis of data was completed in November 2018. Patients were referred by their practitioner to the study, and eligible patients were enrolled by trained research staff using written informed consent procedures approved by the Johns Hopkins Institutional Review Board. Although enrolled participants were encouraged to engage a parent or other supportive adult in their life, a waiver of parental consent was granted by the institutional review board because patients were being treated for an STI and Maryland state law provides for minor consent to counseling and treatment for STIs. For patients younger than 18 years who were enrolled in the intervention, additional written parental consent was required at the time of the home visit to conduct the intervention in the home. This study followed the Consolidated Standards of Reporting Trials (CONSORT) reporting guideline.

### Eligibility Criteria

**Figure 1.**
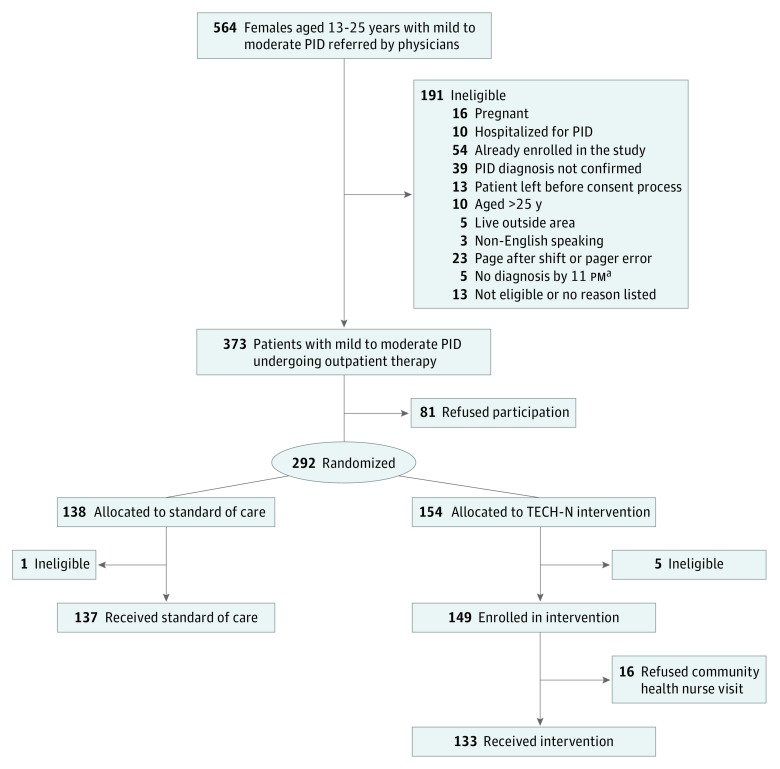
CONSORT Flow Diagram PID indicates pelvic inflammatory disease; TECH-N, technology enhanced community health nursing. ^a^Recruitment hours were 8 am to 11 pm.

Patients were eligible for this single-blinded randomized clinical trial if they were 13 to 25 years of age, had a diagnosis of mild to moderate PID, had a disposition plan for discharge to outpatient treatment at home instead of hospitalization, resided in the local metropolitan area, were willing to be visited by a community health nurse and participate in text-messaging communication if randomized to the intervention, and were willing to be randomized during the informed consent process (Figure 1). Patients who were pregnant, hospitalized for PID, already enrolled in the study and had rediagnosed PID, receiving care for a sexual assault, or unable to communicate with staff because of cognitive, mental, or language difficulties were ineligible for study participation.

### Study Procedures

At baseline, participants completed an audio computer-assisted self-interview and were randomized to intervention and standard of care control groups based on a computer-generated block randomization sequence that was generated by the study’s data analyst (S.C.) and sealed in an envelope until sequential enrollment.^16^ Although PID is a polymicrobial illness, the hallmark of assessment and treatment of patients is based on the effective treatment of *N gonorrhoeae* and *C trachomatis* as commonly known pathogens. All participants provided self- or practitioner-collected vaginal swabs to test for STIs, including *N gonorrhoeae* and *C trachomatis* infections, and received a full course of medications per the CDC STI treatment guidelines^7,8^ that were dispensed through their practitioner with routine discharge instructions. All patients received a single intramuscular injection of 250 mg of ceftriaxone per their routine clinical care and received twenty-eight 100-mg doxycycline tablets to take 1 tablet orally twice daily for 14 days. Other medications were available when practitioners requested them (eg, 500 mg of metronidazole twice daily for 14 days) to dispense at discharge with doxycycline. All participants were provided with standard discharge instructions per institutional PID treatment guidelines, and control participants were asked to arrange a 72-hour visit with their primary care practitioner or with the Title X program offered through the institutional adolescent and young adult clinic.

### Intervention

Intervention participants received daily, automated text-message reminders for 2 weeks and then weekly booster messages provided through the Health Cloud SMS mobile communications platform (ReifyHealth LLC) for 1 month using their personal mobile phone or a prepaid, disposable mobile phone with text-messaging capacity provided by the study to patients without mobile phone access at the time of enrollment for use during the study. The daily messages reminded patients to take their medications and prompted patients to provide data about how many scheduled doses they had consumed each day with a tailored automated message to affirm and encourage adherence to the treatment regimen based on their responses (eTable 1 in Supplement 2).

A community health nurse interventionist trained to deliver the PID-specific, short-term clinical follow-up visit that included a complete clinical assessment with abdominal examination and a 20-minute skills-based sexual risk reduction and condom negotiation counseling session using the Sister-to-Sister Teen Intervention^17,18^ also visited intervention participants within 5 days of the enrollment visit to complete the CDC-recommended short-term clinical reassessment. This time frame was set up based on standards set by other national PID trials.^19^ Alternative community sites (eg, local school-based health centers and health department clinical sites) for follow-up visits were determined in consultation with participants who did not believe that they were able to have the visit at home safely. The outreach worker saw all patients for research study visits at 14, 30, and 90 days after enrollment. During these visits, research staff conducted interviews and STI specimen collection. All study participants with positive results were contacted by a research nurse practitioner, notified of their positive test result, and referred for treatment to the Title X clinic per the protocol. Cases of *N gonorrhoeae* and *C trachomatis* infection were reported to the local health department per Maryland state law. The TECH-N research staff encouraged partner notification and treatment while providing education, resources, and no-cost clinical care through the institutional Title X program for patient and partner treatment.

### Remuneration

Participants received $10 at enrollment, $10 for each completed face-to-face research visit (14, 30, and 90 days), and $10 for each STI sample (n = 3) provided for the study. As noted earlier, participants also received oral discharge medications for the 14-day treatment period at no cost. Finally, participants who notified the team of interim changes in the contact information (eg, mobile phone number, home address) received $5 in additional remuneration.

### Measures

Core measures used for this analysis included the recruitment, retention, and intervention delivery data, sociodemographics, and sexual and reproductive history data from the audio computer-assisted self-interview, self-reported adherence measures (partner notification and treatment, sexual abstinence, and self-medication adherence from the 14-day visit). Biological outcomes under study were *N gonorrhoeae* and *C trachomatis* test results at 0, 30, and 90 days. The Johns Hopkins Hospital laboratory performed baseline *N gonorrhoeae* and *C trachomatis* testing. Subsequent specimens were processed by the Johns Hopkins University International STD Research Laboratory. Vaginal specimens were tested according to the Aptima Combo 2 (Hologic Inc) package inserts for *N gonorrhoeae* and *C trachomatis*.

### Statistical Analysis

#### Sample Size

Sample size calculations were based on the longitudinal data from a prior research trial^13^ focused on additional STIs and PID after an initial diagnosis of PID. Assumptions for this study were STI positivity of 25% at 3 months, recruitment targets of 350 based on hospital billing and coding data, and 30% attrition, giving us an effective sample size of 245 participants. With use of these assumptions, our initial power calculations suggested that we would have 80% power to detect a relative risk of 0.6.^13^

#### Data Analysis

This study compared the efficacy of the intervention with that of the standard of care using an intention-to-treat analysis. We used logistic regression analyses to examine the primary outcome of probability of STI at 90 days after diagnosis of PID and to examine the secondary 14-day self-care adherence outcomes associated with PID. Generalized estimating equations were used to determine whether the intervention group had a time-related decrease in the odds of recurrent or incident STIs during the 90-day follow-up period with generalized estimating equations and a logit link function given the following formula:
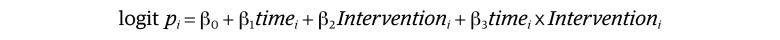
In the formula, *p_i_* indicates the probability that a participant’s *C trachomatis* or *N gonorrhoeae* test result is positive, β_0_ indicates the log odds of *C trachomatis* or *N gonorrhoeae* infection at baseline in the control arm, β_1_ indicates the log odds ratio of infection 1 month after baseline compared with baseline in the control arm, β_2_ indicates the log odds ratio of infection in the TECH-N arm compared with the control arm at baseline, and β_3_ indicates the difference in the log odds of infection over time between the control and TECH-N arms, such that the log odds ratio of infection at time 1 month in the TECH-N arm is β_1_ + β_3_ compared with baseline. The variable of interaction between time and intervention β_3_ is the difference in the log odds of STI positivity for every unit increase in time for the intervention group given the differential in baseline *C trachomatis* test results. All analyses were conducted in R, version 3.3.0 (R Project for Statistical Computing). Two-sided *P* < .05 was considered to be statistically significant.

## Results

### Recruitment Outcomes

Of the 564 individuals approached for the study, 373 were eligible and 292 were enrolled in the trial for a 78% acceptance rate. Six individuals were excluded after enrollment because they did not meet the enrollment criteria (eg, hospitalized after doxycycline treatment, later revealed an out-of-state address), resulting in a final active enrollment of 286 participants (149 in the intervention group and 137 in the control group; mean [SD] age, 18.8 [2.5] years; 268 [93.7%] African American). Patient demographics are given in Table 1. Of the 154 enrolled participants in the intervention, 16 refused the community health nurse visit. The effective intervention delivery rate achieved was 89.6% (138 of 154 patients), and 90.9% (260 of 286 patients) of the effective sample was retained at 3 months (Figure 1). Only 5 individuals required the use of the study-provided disposable mobile phone.

**Table 1.** Baseline Demographics by Intervention Group and for 286 Study Participants^a^

Demographic	Overall	Intervention	Control	*P* Value^b^
Age, mean (SD), y	18.8 (2.5)	18.7 (2.5)	18.9 (2.5)	.64
Race/ethnicity				
African American	268/286 (93.7)	140/149 (94.0)	128/137 (93.4)	.64
Hispanic	6/286 (2.1)	2/149 (1.3)	4/137 (2.9)	
White	7/286 (2.4)	4/149 (2.7)	3/137 (2.2)	
Other	5/286 (1.7)	3/149 (2.0)	2/137 (1.5)	
Insurance				
Medicaid	247/286 (86.4)	131/149 (87.9	116/137 (84.7)	.38
Private	20/286 (7.0)	11/149 (7.4)	9/137 (6.6)	
Self-pay	19/286 (6.6)	7/149 (4.7)	12/137 (8.8)	
Age at sexual debut, mean (SD), y	14.8 (1.7)	14.9 (1.9)	14.7 (1.6)	.49
No. of lifetime partners, mean (SD)	6.1/277 (6.8)^c^	5.8/147 (5.9)	6.3/130 (7.6)	.54
Ever diagnosed with STI	162/282 (57.4)^c^	80/148 (54.1)	82/134 (61.2)	.23
Ever been pregnant	152/280 (54.3)^c^	79/146 (54.1)	73/134 (54.5)	.95
STI at baseline				
Chlamydia	70/271 (25.8)^c^	45/139 (32.4)	25/132 (18.9)	.01
Gonorrhea	27/271 (10.0)^c^	11/139 (7.9)	16/132 (12.1)	.25
Chlamydia or gonorrhea	82/273 (30.0)^c^	48/140 (34.3)	34/133 (25.6)	.12

Abbreviation: STI, sexually transmitted infection.

^a^ Data are presented as number/total number (percentage) of patients unless otherwise indicated.

^b^ Significance determined by the *t* test or χ^2^ test where appropriate to compare percentages between groups.

^c^ Number of observations vary because of participant nonresponse, sample leakage, or indeterminate diagnostic results.

### Baseline STI Rates

Participants had an overall mean (SD) age at sexual debut of 14.8 (1.7) years and 6.1 (6.8) lifetime sexual partners. Prior STI morbidity and pregnancy were high, with 162 patients (56.6%) reporting a history of STIs and 152 (53.1%) having a history of pregnancy. Although the study groups were demographically similar, indicating successful randomization, the intervention group had a higher baseline prevalence of *C trachomatis* infection (45 of 139 [32.4%] vs 25 of 132 [18.9%], *P* = .01) (Table 2).

**Table 2.** Summary of Adherence Measures and STI Diagnoses at 90 Days After Enrollment Day by Intervention Arm

Adherence Measure	Patients, No./Total No. (%)			*P* Value^b^
	Overall^a^	Intervention	Control	
Follow-up visit within 72 h	151/262 (57.6)	131/139 (94.2)	20/123 (16.3)	<.001
All medication taken	112/260 (43.1)	52/137 (38.0)	60/123 (48.8)	.08
Abstinence	209/260 (80.4)	107/137 (78.1)	102/123 (82.9)	.33
Partner notification	237/260 (91.2)	124/137 (90.5)	113/123 (91.9)	.70
Partner treated^c^	116/1990 (61.1)	55/99 (55.6)	61/91 (67.0)	.14
90-d STI results				
Chlamydia^d^	9/259 (3.5)	4/134 (3.0)	5/125 (4.0)	.66
Gonorrhea^d^	11/259 (4.2)	2/134 (1.5)	9/125 (7.2)	.03
Chlamydia or gonorrhea	19/260 (7.3)	6/135 (4.4)	13/125 (10.4)	.07

Abbreviation: STI, sexually transmitted infection.

^a^ Number of measurements vary slightly because of participant nonresponse, sample leakage, and indeterminate diagnostic results.

^b^ Significance determined by the χ^2^ test.

^c^ Number of responses vary because of unknown status of partner treatment.

^d^ Significance determined by the Fisher exact test.

### Adherence Measures and STI Diagnoses at 90 Days

Overall, 262 participants (91.6%) had successful 14-day follow-up outreach study visits. The overall short-term follow-up visit rate was 57.6% (151 of 262 patients); 43.1% (112 of 260 patients) completed all medications, 80.4% (209 of 260) abstained from sexual intercourse during treatment, 91.2% (237 of 260 patients) notified their partner for treatment, and 61.1% (116 of 190 patients) reported successful partner treatment (Table 2). Intervention participants were significantly more likely to receive the recommended short-term follow-up (131 of 139 [94.2%]) compared with the control group (20 of 123 [16.3%]; adjusted odds ratio, 86.3; 95% CI, 34.9-213.5; *P* < .001); however, no significant differences were found in medication completion, abstinence, or partner notification or treatment (Table 3).

**Table 3.** Logistic Regression Results for Measures of Adherence^a^

Outcome	Unadjusted		Adjusted^b^	
	Odds Ratio (95% CI)	*P* Value	Odds Ratio (95% CI)	*P* Value
Follow-up visit within 72 h	84.3 (35.7-199.2)	<.001	86.3 (34.9-213.5)	<.001
All medication taken, yes or no	0.6 (0.4-1.1)	.08	0.6 (0.4-1.1)	.08
Abstinence	1.0 (0.9-1.1)	.33	1.0 (0.9-1.1)	.35
Partner notification	0.8 (0.4-2.0)	.70	1.0 (0.4-2.4)	.97
Partner treated	0.6 (0.3-1.1)	.14	0.6 (0.3-1.2)	.15

^a^ Odds ratios are for the chance that a participant receiving the intervention will have followed recommended treatment compared with those in the control arm.

^b^ Adjusted for age, insurance, debut age, number of lifetime partners, pregnancy history, and baseline sexually transmitted infection positivity (any sexually transmitted infection).

### 90-Day STI Outcomes

**Figure 2.**
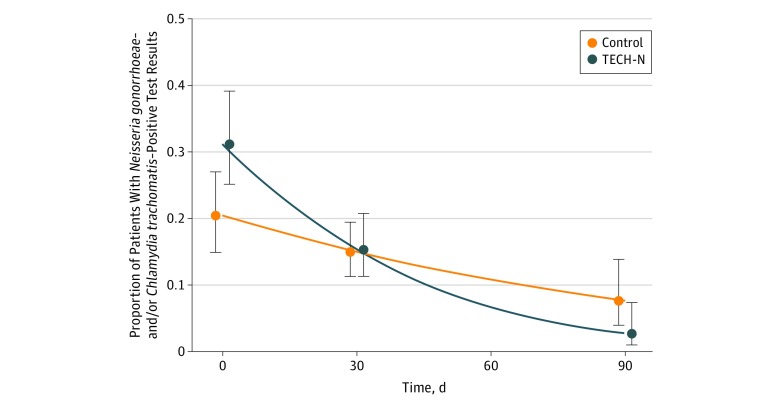
Patients Testing Positive for *Neisseria gonorrhoeae* and/or *Chlamydia trachomatis* Over Time as Determined With Generalized Estimating Equations by Intervention Group Error bars indicate the 95% CIs at each follow-up time point estimated with the Wald test. TECH-N indicates technology-enhanced community health nursing.

Absolute reductions in the prevalence *N gonorrhoeae* infection were greater in the intervention group than in the control group (1.5% [2 of 134 patients with infection at 90 days] vs 7.2% [9 of 125 patients with infection at 90 days], *P* = .03). The rate of recurrent or incident *N gonorrhoeae* and *C trachomatis* infection at 90 days was lower in the intervention group, but this finding did not reach statistical significance (6 of 135 [4.4%] vs 13 of 125 [10.4%], *P* = .07). The *N gonorrhoeae* and *C trachomatis* positivity rate decreased over time in both groups; however, the differential rate of decrease was higher in the intervention group (eTable 2 in Supplement 2). Generalized estimating equations were used to examine the change over time, accounting for the difference in baseline *C trachomatis* prevalence. The TECH-N participants had a significantly higher differential rate of decrease compared with the control group at 90 days (48 of 140 [34.4%] to 6 of 135 [4.4%] compared with 34 of 133 [25.6%] to 13 of 112 [10.4%], *P* = .02) (Figure 2).

### Condom Use

Condom use instruction was a critical part of the intervention. The overall use increased from 17.2% (48 of 279 patients) to 33.2% (86 of 259 patients) in study participants, and higher condom use was observed at 3 months in the intervention arm compared with the control group (23 of 146 [15.7%] to 49 of 134 [36.6%] vs 25 of 133 [18.8%] to 37 of 125 [29.6%]), but this difference did not reach statistical significance (*P* = .10) (eTable 3 in Supplement 2).

## Discussion

The TECH-N group had a significantly greater decrease in *N gonorrhoeae* and *C trachomatis* infection during 90 days than the standard of care control group. The TECH-N intervention was also efficacious for improving adherence to the CDC-recommended short-term clinical follow-up visit after PID among adolescents and young adults 13 to 25 years of age.

These findings fill a particularly significant gap in the field given that more than half of the patients in the sample had prior STIs, which has the potential to compound the risk for sequelae in the context of recurrent disease. Many women seek services in emergency department settings but require close follow-up after their diagnosis to ensure recovery and to address related issues, such as STI and HIV risk reduction and contraceptive counseling. Community health nursing has been an effective strategy for treating adolescents and young adults who require ongoing support for management of clinical issues, such as asthma, teen pregnancy, and parenthood.^20,21,22^ Working with adolescents and young adults with complex STIs in the community must be navigated thoughtfully to preserve confidentiality, while also providing needed services. This study demonstrates that the TECH-N approach is safe and efficacious for adolescents and young adults with mild to moderate PID.

The use of technology as a part of the TECH-N design to support intervention patients is also in line with the notion of reaching adolescents where they are. National data demonstrate that 95% of adolescents have a smartphone and are online almost constantly.^23^ Furthermore, study findings suggest that the digital divide has closed for low-income urban youth owing to smartphone access. Only 3% of intervention participants required a study-provided mobile phone at enrollment. Technology access via a mobile phone is consistent with our prior research demonstrating that more than 95% of sexually active adolescent and young adult women have access to mobile phones with text messaging and internet access.^24^ This work provides information for the potential development of interventions that leverage technology for care management and decision support for serious conditions for which confidentiality and personalized support are essential.

Pelvic inflammatory disease is a common condition that can affect the reproductive health trajectory of female adolescents and young adults. There has been a shift in the CDC guidelines over time to deliver care to youth with mild to moderate disease in outpatient settings in response to the outcomes from the PEACH trial.^19^ Although patients were captive in the hospital during the PEACH trial, inpatient care did not include individualized risk-reduction counseling for STIs, HIV infection, and pregnancy using evidence-based approaches and it did not account for most care being self-provided in the outpatient setting. To our knowledge, this was the first intervention trial designed to improve key adherence measures and health outcomes for female adolescents and young adults in the outpatient setting.

Treatment of PID requires a complex set of behaviors to ensure not only that the patient and her sexual partner are adequately treated but also that future behavior is appropriately managed, with careful sex partner selection and condom negotiation behaviors. Although this single intervention was not able to adequately address all the required behavioral changes for patients, participation in the TECH-N intervention significantly increased the receipt of the CDC-recommended short-term clinical follow-up care and reduced the rate of acquisition for *N gonorrhoeae* and *C trachomatis* infection, 2 key organisms associated with PID and its sequelae. Given that the recommendation for follow-up care in the office within 72 hours is standard practice, these data suggest that community health nursing may be a more effective strategy for actual care delivery; less than 20% of the control participants sought the recommended short-term clinical services available at no cost to them.

### Limitations

The findings from this research must be considered in light of several general limitations. This sample was a relatively homogeneous group of mostly low-income, African American adolescents and young adults who were cared for within a single institution and community. Findings may not be generalizable to other institutions or communities. However, there are significant STI and PID disparities facing young, African American women; thus, work with this population is important from a public health perspective. Similar to the PEACH trial, the potential confounding effects of socioeconomic status and race/ethnicity are difficult to parse because of the PID burden borne by low-income youth facing a high community burden of STIs. Texting for adherence reports was only used for intervention participants; thus, we used a self-report for the medication adherence variable. The outreach worker who collected the 2-week, 30-day, and 90-day data was a single individual and was not blinded to group assignment information. It is possible that interactions with the outreach worker may have influenced the longitudinal behavior of adolescents in the control group, but this individual did not interact with participants during the 14-day intervention period. This role requires a highly specialized skill set for field-based operations and represents an important attention control and mechanism for effective data collection in vulnerable populations that are difficult to engage in research involving randomized clinical trials. In addition, because of the delay in detection of *C trachomatis* as the causative agent for PID and the necessity of immediate action related to intervention participants, randomization was not stratified by *N gonorrhoeae* and *C trachomatis* diagnosis and an imbalance in baseline *C trachomatis* infection was observed. This delay in access to testing results is standard of care because federally approved point of care testing for *C trachomatis* with results within the visit time frame is not currently available. This study had active outreach with all participants at 14, 30, and 90 days, and there was potential for a positive influence on control group behavior.

## Conclusions

The TECH-N intervention showed sufficient success for delivery of the CDC recommendations for interim care and short-term reduction in STI acquisition and should be considered as a potential enhancement of standard of care approaches for female adolescents and young adults with mild to moderate PID in urban communities facing significant STI disparities. Training the local pool of home and community health nurses to complete the CDC-recommended post-PID assessments and risk reduction counseling in the field may optimize outcomes by providing essential acute care management support and reducing subsequent *N gonorrhoeae* and *C trachomatis* infections. A cost-effectiveness evaluation of such a community-level scale-up is warranted.

## Notes

Supplement 1. **Trial Protocol**

Supplement 2. **eTable 1.** Sample Automated Text Support Messages Sent to 286 Women With Pelvic Inflammatory Diseases Enrolled in the Technology-Enhanced Community Health Nursing (TECH- N) Trial **eTable 2.** Predicted Change From Baseline STI Positivity at 30- and 90-Day Follow-up Among 286 Women With Pelvic Inflammatory Diseases Enrolled in the Technology-Enhanced Community Health Nursing (TECH- N) Trial **eTable 3.** Baseline and 3-Month Condom Use at Last Sexual Intercourse, by Intervention Group and for All Study Participants

Supplement 3. **Data Sharing Statement**
