# Supplementary material for: Efficacy of a Technology-Enhanced Community Health Nursing Intervention vs Standard of Care for Female Adolescents and Young Adults With Pelvic Inflammatory Disease: A Randomized Clinical Trial
Source: JAMA Netw Open. 2019 Aug 7;2(8):e198652. doi: 10.1001/jamanetworkopen.2019.8652 (PMC6686980; doi:10.1001/jamanetworkopen.2019.8652)
Supplement: Supplement 1. — Trial Protocol [file jamanetwopen-2-e198652-s001.pdf]

JHM IRB - eForm A

- **Use the section headings to write the JHM IRB eForm A, inserting the appropriate material in each. If a section is not applicable, leave heading in and insert N/A.**
- **When submitting JHM IRB eForm A (new or revised), enter the date submitted to the field at the top of JHM IRB eForm A.**

****

1. Abstract

Pelvic Inflammatory Disease (PID) remains a serious reproductive health disorder and disease rates remain unacceptably high among minority adolescent girls and young adult women. Each episode of this upper reproductive tract infection, usually caused by a sexually transmitted infection (STI), increases the risk for multiple sequelae including tubal infertility, ectopic pregnancy, and chronic pelvic pain (CPP). Care for PID has shifted from the inpatient to the outpatient setting; however, our age-stratified analysis of national data shows that adolescents continued to have high rates of repeat STIs and CPP, regardless of treatment strategy. Our local clinical trial data has also shown that urban adolescents have poor adherence to the 3 day clinical follow-up visit recommended by the Centers for Disease Control and Prevention (CDC), are at high risk for STI at the 90-day STI re-screening visit, and that brief interventions in the acute care setting have positive, but modest effects on adherence to self-management behaviors. Inpatient treatment for PID is expensive without incremental increases in effectiveness when compared with outpatient treatment so developing outpatient strategies to improve short and long-term reproductive health outcomes, including recurrent STI and PID, are warranted. Community health nurse (CHN) interventions have been shown to increase access to appropriate resources enhance health care utilization and promote risk-reducing behavior among adolescents. Recent research has also shown that use of short messaging service (SMS) messaging can enhance clinical care by improving attention to medical visits, medication adherence, and communication with the health care team. The primary aim of this project is to compare the effectiveness of a technology-enhanced community health nursing intervention (TECH-N) intervention to an optimized standard of care control group using randomized trial design. We hypothesize that repackaging the recommended CDC-PID follow-up visit using the TECH-N will be cost-effective compared with standard of care and reduce rates of short-term repeat infection by improving adherence to PID treatment and reducing unprotected intercourse. We have demonstrated that our team of trained community-oriented health professionals can follow adolescent girls with PID in the community and that utilizing CHNs to optimize treatment in the post-PID period is feasible and acceptable to adolescents and parents in urban communities. Our pilot using SMS communication also demonstrates that urban adolescent girls have access to cell phone technology, eagerly accept SMS as a communication strategy, and respond to health provider queries for sexual health maintenance support. We propose to enroll 350 young women 13-25 years old diagnosed with PID in Baltimore and randomize them to receive the TECH-N intervention which includes CHN clinical support using a single post-PID face-to-face clinical evaluation that incorporates an evidence based STI prevention curriculum and SMS

communication support during the 30-day period following diagnosis (intervention group) or the standard of care (control group).

2. Objectives

To compare the effectiveness of a PID intervention called Technology Enhanced Community Health Nurse (TECH-N) compared to a control group defined as optimized standard of care using a randomized control trial (RCT) among urban adolescents diagnosed with PID.

Hypothesis 1: Adolescents in the TECH-N intervention group will have a better rate of short term (3-5 day) clinical follow-up compared with adolescents in the control group.
Hypothesis 2: Adolescents with PID in the TECH-N intervention group will have a lower rate of recurrent STIs at 90-days compared with the adolescents with PID in the control group.
2. To compare the cost-effectiveness of TECH-N as compared to control (optimized standard of care) for management of PID in the outpatient setting.
Hypothesis 3: The TECH-N intervention will be more cost-effective than the optimized standard care control approach.
Hypothesis 4: The TECH-N intervention will be more cost-effective than hospitalization using modeled parameters of existing cost estimates among patients with mild to moderate disease.

3. **Background** (briefly describe pre-clinical and clinical data, current experience with procedures, drug or device, and any other relevant information to justify the research)

Pelvic Inflammatory Disease (PID) remains a serious reproductive health disorder and disease rates remain unacceptably high among minority adolescent girls and young adult women. Each episode of this upper reproductive tract infection, usually caused by a sexually transmitted infection (STI), increases the risk for multiple sequelae including tubal infertility, ectopic pregnancy, and chronic pelvic pain (CPP). Care for PID has shifted from the inpatient to the outpatient setting for cost containment and in response to efficacy data from the Pelvic Inflammatory Disease Evaluation and Clinical Health (PEACH) study that demonstrated similar fertility outcomes for women treated in the inpatient and outpatient setting. Unfortunately, our age-stratified analysis of the PEACH study data shows that adolescent and young adult women continued to have high rates of repeat STIs and CPP, regardless of treatment strategy. Twenty percent of women less than 25 years experienced a repeat or persistent STI at 30 days and/or repeat PID over the 7 year follow-up period was associated with a 5-fold risk of CPP. Our local clinical trial data has also shown that urban adolescents and young adults have poor adherence to the developmentally appropriate 3 day clinical follow-up visit recommended by the Centers for Disease Control and Prevention (CDC), are at high risk for STI at the 90-day STI re-screening visit, and that brief interventions in the acute care setting have positive, but modest effects on adherence to self-management behaviors. Previous research demonstrates that inpatient treatment for PID is expensive without incremental increases in effectiveness when compared with outpatient treatment. Our work and that of others suggest that additional outpatient cost-effective PID health care supports are needed for this vulnerable population to improve short and long-term reproductive health outcomes, including recurrent sexually transmitted infection and PID.

Prior research has demonstrated that community health nurse (CHN) interventions can increase access to appropriate resources enhance health care utilization and promote risk-reducing behavior. We propose that integrating a technology component conducted by the CHN will increase appeal to adolescent females. Cell phone penetration among youth in the United States has increased dramatically such that teens rely on text messaging in their daily lives. Recent research has shown that use of SMS messaging enhances clinical care by improving attention to medical visits, medication adherence, and communication with the health care team. Our pilot data of a text messaging intervention for reproductive health clinical reminders has demonstrated that use of cell phones to assist urban adolescents residing in high STI prevalent communities with self-care is both highly acceptable and feasible.

We hypothesize that repackaging the recommended CDC-follow-up visit using a technology-enhanced community health nursing intervention (TECH-N) with integration of an evidence-based STI prevention curriculum will reduce rates of short-term repeat infection by improving adherence to PID treatment and reducing unprotected intercourse and be more cost-effective compared with outpatient standard of care (and hospitalization). We have demonstrated that our team of trained community-oriented health professionals

can follow adolescent girls with PID in the community and that utilizing CHNs to optimize treatment in the post-PID period is feasible and acceptable to adolescents and parents in urban communities. Our pilot using SMS communication also demonstrates that urban adolescent girls have access to cell phone technology, eagerly accept SMS as a communication strategy, and respond to health provider queries for sexual health maintenance support. We propose to enroll 350 young women 13-21years old diagnosed with PID in Baltimore and randomize them to receive CHN clinical support using a single post-PID face-to-face clinical evaluation and SMS communication support during the 30-day period following diagnosis (intervention group) or optimized standard of care (control group).

4. Study Procedures

a. Study design, including the sequence and timing of study procedures (distinguish research procedures from those that are part of routine care).

Overview of Study Design

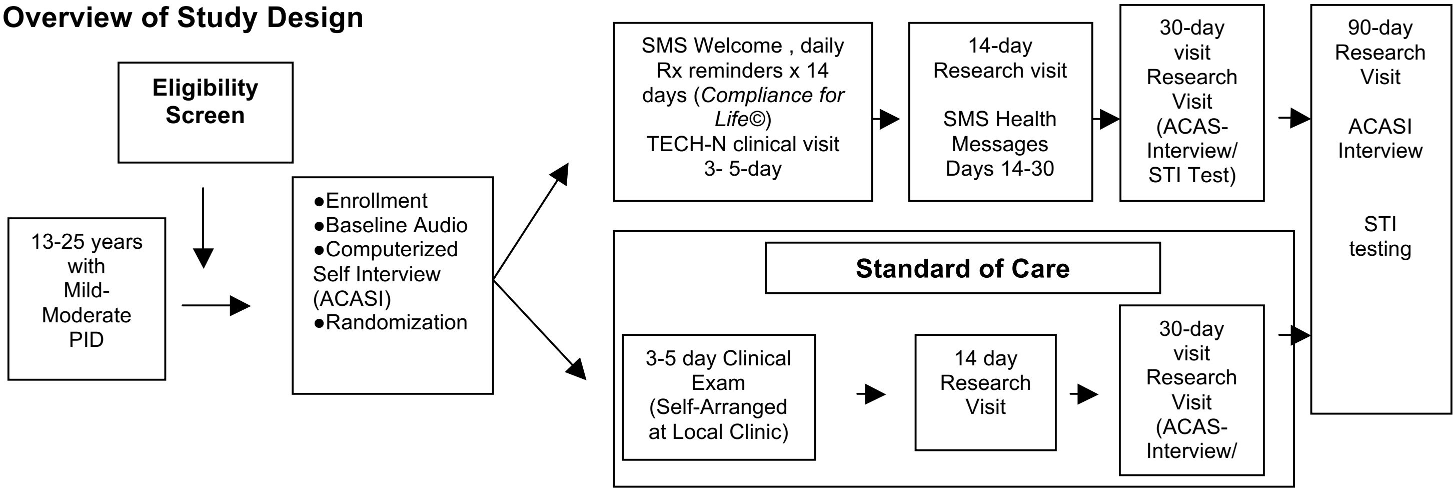

1. Setting: This research study will be conducted in Baltimore, Maryland. Maryland currently ranks 12th in the nation for incident infections with Neisseria gonorrhea (GC) and Chlamydia (CT) among its citizens and there are disproportionate rates of infection among individuals residing in Baltimore.[87] The Johns Hopkins Hospital, is geographically located in one of the highest STI prevalence areas in the city and institutional providers diagnose approximately 150 cases of PID to adolescents 21 years of age or younger each year.

2. Participants and Recruitment: 350 adolescents, aged 13-25 years with mild-moderate PID will be recruited from Johns Hopkins Hospital System sites at the time of diagnosis by the TECH-N study staff. Johns Hopkins Hospital clinical sites include the Pediatric Emergency Department and the Harriet Lane General Pediatric and Adolescent Clinics.

3. Enrollment, Consent, and Treatment: Recruiters will be onsite in each clinical setting to recruit patients diagnosed with PID. Flyers will be placed in physician and nursing work areas in all recruitment sites as a reminder of ongoing study recruitment. Patients will be screened for eligibility criteria, and study follow-up visits visit will be scheduled. At enrollment, a detailed review of the study with informed consent will be performed by the research staff. All patients who agree to participate will then complete an audio computerized assisted self-interview (ACASI) to collect baseline information and be randomized to a research arm. Study forms will be faxed to the project director at the time of enrollment and the ACASI interview will be automatically uploaded to a secure server for warehousing in the central database. After completion of initial study procedures and instruction, patients will receive remuneration of $10 and a 14-day course of 100 mg doxycycline tablets to be dispensed by the clinician prior to discharge according to hospital policy. Adolescents who are eligible for the trial and assigned to the intervention arm, but do not have a cell phone will be provided temporary use of a cell phone during the 1 month during which messages are sent. We anticipate that this will be a small number of adolescents given that CTIA asserts that

there is 93% cell phone penetration in the United States [56] and 88% of the girls approached for our Depo-Text study had a cell phone for their personal use.

4. Randomization: Following completion of the baseline ACASI survey, each adolescent will be randomly assigned to either the TECH-N or control group using a permutated block design. A permutated block design will be used to ensure group balance based on JHH sites at the end of the trial and period balance. The period balance will help to minimize any systematic differences between patients enrolling at different times during the study.[88]

5. Intervention

TECH-N

The TECH-N intervention involves contact and follow-up by a CHN trained in disease intervention protocols, clinical assessment of PID, and STI prevention case management so that they can effectively deliver the recommended CDC follow-up in the field. The nurse will visit the adolescent at 3-5 days. While the CDC recommends follow-up within 72 hours, our work suggests that 72 hours is an unrealistic timeline for tracking high risk girls in the community.[18] The mean time to follow-up in our pilot study was 5.6 days (See Section 3.C.6) and is consistent with the follow-up evaluations timeline used for the PEACH trial.[4, 35, 40] Given that only 20% of adolescents are making the follow-ups at all, extending the home visit target to a 3-5 day window is practical, ethical, and justifiable. The TECH-N intervention will be enhanced by the use of the SMS messaging system. Adolescents in the TECH-N arm will be enrolled in the online ***Health Cloud, SMS***© that will deliver a welcome message, a prompt to schedule the appointment for the 3-5 day follow-up with the TECH-N nursing team, and daily medication reminders for 14 days. After 2 weeks, the patient will also receive positive health reminders 3 times per week for the rest of the month (total 30 days).

The TECH-N is based on the CDC STD Treatment Guidelines and modeled after the Johns Hopkins Children's Center PID Treatment protocol for inpatient nursing staff. Integral to the TECH-N intervention is use of an effective behavioral intervention to reduce STI/HIV risk reduction behaviors. We will use the Sister-to-Sister intervention that has a 20-minute one-on-one module to guide the patient through skill-based risk reduction counseling. This intervention was evaluated with young African American women in an urban community with high STI rates and was found to increase condom use, reduce unprotected intercourse, and reduce new STIs among participants.[68, 69] At the end of the session with the CHN, the patient will be able to state/demonstrate: 1.) Effective pain management; 2.) High understanding of the definition of STIs including the difference between cervicitis and PID, modes of transmission, signs of complications or problems for which they need to seek additional care, and how to prevent future PID episodes; 3.) High understanding of optimal self-care including: a) the date, time, and place of gynecologic follow-up if indicated, b) the contact phone numbers for the TECH-N nurse and an MD provider, c) medication schedule, route, dose, and side effects, d) proper use of male and female condoms e) reportable signs and symptoms (e.g. fever, increasing abdominal pain, nausea/vomiting, or inability to tolerate medication regimen), and f) effective partner communication and negotiation strategies.

While the TECH-N nurse will perform the basic clinical evaluation for patients enrolled in this arm, the TECH-N nurse may determine that additional physician follow-up is indicated. At the end of the visit the TECH-N will determine a plan of action using two conditional algorithms based on the clinical assessment; one for patients requiring immediate attention (e.g. symptomatic worsening abdominal pain) and the other for patients for whom non-immediate follow-up is indicated (e.g. patient desires ongoing hormonal contraceptive method) The TECH-N nurse will also be given referrals resources to assist patients who may have other issues identified during the visit (child protection, housing, substance abuse in a family member, and/or other adolescent stressor, medical or mental health co-morbidity).

We are using professional CHNs as the TECH-N, comparable to the STI case management model, rather than paraprofessionals. The TECH-N nurse will be trained on clinical assessment after

PID, disease intervention protocols including partner notification treatment/accessing community resources for partner care, STD medication treatment, CDC-supported Sister to Sister STI prevention protocol, use of contingency contracts with adolescents, and TECH-N protocols. The TECH-N nurse will also be an expert on the updated CDC PID Treatment guidelines. Despite the increase in cost of a professional nurse, consistent evidence indicates that paraprofessional home visiting interventionists produce smaller effects compared to professionals[89] and low-resource patients benefit most from nurse visit interventions.[90] Our preliminary studies indicate that improved communication between an adolescent and her partner occurs with the support of a CHN who can educate and motivate adolescents with an STI, provide expertise on STI management, and encourage higher level service provision by primary care providers when problems are identified in the community-based environment. [79]

Control Condition

In the control condition, patients will receive standard of care. The Johns Hopkins Children's Center JHCC has optimized PID care to ensure delivery per CDC guidelines. All clinical sites use electronic medical records and ordering systems to facilitate uniform care. Dr. Trent has developed specialized institutional PID order sets to ensure integration of standard management protocols into usual clinical care. After completion of study enrollment procedures, ACASI baseline survey, and collection of a vaginal swab for Mycoplasma genitalium (not usually sent as a part of standard practice), the clinical provider will dispense the study-supplied doxycline and discharge the patient with instructions for self-care using the JHH PID resources. The team will have access to other medication treatment options, but in the last 5 years we have not encountered a patient who could not use doxycycline due to medication allergy. Per institutional policy, patients with PID seen in the JHCC PED may follow-up in the JHH Harriet Lane Adolescent/Young Adult Clinic for the 72 hour visit. Patients can be seen in this setting for reproductive health services regardless of ability to pay through the clinic's Title X program. Patient's who use this service are encouraged to subsequently resume care with their primary care provider given the limitations on the type of care that can be provided through this program. Patient will return to the hospital for research visits at 14 days, 30 days, and 90 days. STI testing will be performed at the 30- and 90 -day study visits.

Outreach worker will contact all patients for a brief quarterly interview about their reproductive health status will contact via phone and/or in person ACASI. The current consent form allows research team to contact patients for future related studies. Patients who have already been enrolled will be re-contacted and verbal consent for ongoing follow-up for up to 36 months after re-enrollment.

6.Quality Control

a. Training of Personnel. All research staff, P.I. and co-investigators received training in Protection of Human Subjects in research studies. Research assistants and interviewing staff will be trained by Dr. Trent on the recruitment and consent protocol, interview surveys and confidentiality of data collection. The TECH-N CHN will conduct all TECH-N visits and has expertise in managing STIs in the local health department and in the home/community. The TECH-N will be trained by Drs. Butz and Trent for delivery of the intervention.

b. Recruitment Monitoring: Research staff will be available in on site order to make patient referral and recruitment as easy as possible. Research staff will monitor weekly diagnosis reports to determine the number of eligible patients and to ensure that none are missed. Ongoing communication between Dr. Anders and Trent will enable the team to identify and address barriers to referral/recruitment. Research staff will also record all patients approached for study participation and document all referrals and track numbers of eligible participants, those who consent to participate and refusals.

c. Retention Monitoring: Detailed contact information will be obtained from the patient at the time of the initial recruitment, including home and work numbers, cell phone numbers, email addresses,

school attended, and the names of 2 individuals who will always know their whereabouts and can be contacted to locate them. Participants are also advised to call into the study office for changes in their living environment and will receive a small $5 gift card incentive for notification of address change. While we are seeking girls residing in Baltimore Metropolitan Area at the time of diagnosis, we will continue to follow subjects for study visits as long as they continue to reside in the Baltimore Metropolitan Area that includes surrounding counties.

d. Treatment Fidelity: Dr. Arlene Butz who has significant expertise in community health nursing interventions and who has worked with Dr. Trent on several projects including the NANA feasibility trial will train and supervise the TECH-N staff to deliver the intervention as designed. She will also meet weekly with project nursing staff to ensure that they understand study procedures and can use effective strategies to solve problems in the field that maintain staff safety, prevent deviation from protocol, and ensure subject retention in the TECH-N arm of the trial. Video recordings of the first ten visits and thereafter 10% of TECH-N visits will be reviewed by Drs. Butz and Trent for consistency in the TECH-N delivery of specific PID education and management. At any time that an inconsistency in the delivery of protocol is noted, retraining of the TECH-N CHN will occur. Dr. Trent will provide medical supervision of the TECH-N intervention and serve as liaison between the TECH-N nurse and community PCPs. We will also integrate the fidelity checks included with the Sister to Sister intervention according to the field guide. Weekly meetings will be held between the P.I., co-investigators and research staff to review recruitment and data collection.

Cross contamination between groups will be minimized because of the nature, vehicle, and field location of the intervention. Patients may receive additional advice by their doctor at the 3-5 day follow-up visit that assists them in completing the course of medication and other actions involved in self-care, but this will be evenly distributed in both groups due to the study design. We also assess patient satisfaction of the TECH-N follow-up at the 30-day visit, track the number, purpose, and outcome of TECH-N contact between visits, and track scheduled contact behavior.

Study Measurements

a. Recruitment Screener: Includes collection of basic demographic data and the outcome of recruitment effort so that we can keep an accurate account of referral patients who are ineligible and/or refuse to participate in the intervention.

b. Baseline ACASI Survey: We have developed a PID audio-computerized self-interview (ACASI) survey to collect reproductive and sexual health history, perceived barriers to treatment, and self efficacy data from adolescents with PID in acute care settings. The measures on the baseline survey contain: demographics, reproductive and sexual history, PID adherence self-efficacy and perceived barrier scales [17, 71]social provisions scale, [91], Center for Epidemiologic Studies Depression Scale (**CES-D**) as a short measure of depressive symptoms and the short-form survey instrument (SF-12) as a measure of health-related quality of life [42, 92] One question on sexual orientation will be asked to better delineate the risks for this population.

c. Clinical Data: The TECH-N nurse will record contact tracing data and perform a detailed clinical assessment that includes a pain rating scale, abdominal exam, medication usage, supportive care, side effects, activity level, , and patient teaching outcome.. Adolescents in the control arm will be given a short health provider assessment form at enrollment to be completed by the medical professional who re-examines at their 3-5 day clinical follow-up visit. Permission to contact those health providers is requested at the time of enrollment so that project staff can verify self-reported clinical data in the case of unreturned forms. Time to clinical re-evaluation as well as a dichotomous variable indicating completion of the 3-5 day visit will be generated using these measures. Additional clinical data collected at the 2 week research visit will include partner notification, results, and treatment data, sexual abstinence, coping strategies [COPE, Carver 1997] and self-reported medication adherence (pill counts). During this brief face-to face interview, we will also ask questions about partner factors and the participants' perspective the development of preventive PID strategies (e.g. vaccine) and factors affecting their willingness to participate in vaccine research.

d. Follow-up ACASI Surveys: The 30- and 90-day ACASI survey captures interim sexual behavior (current number of sexual partners, change in sexual partners since the 2 week follow-up assessment, change in sexual partner(s), condom use, and contraceptive use, interim diagnosis of STIs and pregnancy, current health status, the adolescent version of the Childbearing Motivation Questionnaire (CBQ) [26], perceptions of partner influences on contraceptive decisions, and participant feelings about their fertility. The ACASI interviews have been used successfully in our previous trial and are easily administered in the field.

e. Cost Data: The costs of administering the different interventions will be based on project records of hours worked, miles traveled, and other supplies with additional amounts for overhead. The primary cost components for patients in both groups include the initial treatment for PID, follow-up therapy related to failure of initial treatment, treatments for recurrent STIs and PID, chronic pelvic pain, ectopic pregnancy, time lost from work/school/household management by patients and parents, travel to PID-related care, and any therapy or evaluation related to concerns about infertility by the patient. We will use these data to estimate the direct (medical costs) and indirect (employment (patient/parent), impairment) costs associated with PID treatment in the TECH-N and control interventions. Health service utilization will be derived from the longitudinal interviews, care utilization data (hospitalizations, emergency department visits, public health clinic visits, primary care provider visits), prescription and over-the-counter medication use, laboratory services, and radiological services (e.g. sonograms). Productivity loss will be derived using the Workplace Productivity and Activity Impairment (WPAI) questionnaire that has been validated as an instrument to collect data on absenteeism and presenteeism both for patients and for care providers. It can also be modified for different diseases and health states without compromising scale properties.[93, 94]

f. Biological Measurements

1. Collection of Biological Samples: Participants will be asked to provide a self-collected vaginal swab (SVS) for Mycoplasma genitalium (MG) and Trichomonas Vaginalis (TV) at baseline to accompany routine testing at the clinical site. Neisseria gonorrhea (GC), Chlamydia trachomatis (CT), and MG will also be obtained at 30 days and 90 days and be processed at The Johns Hopkins University International STD and Biothreat Research Laboratory under the direction of Dr. Charlotte Gaydos. Laboratory tests include:

2. CT and GC assays: The vaginal specimens will be processed and tested according to the APTIMA Combo 2 manufacturer's package inserts for CT and GC. All positive CT and NG APTIMA Combo 2 results from any specimen source will be confirmed using the APTIMA ACT and AGC assays according to instructions provided in the package inserts. Description and performance of these assays have been previously published.[95]

3. MG and TV assays: The APTIMA vaginal swab was tested using the GEN-PROBE transcription mediated amplification (TMA) research assay in the same manner as for the other APTIMA assays. This assay targets MG rRNA and TV rRNA for detection in genital specimens.

4. Notification of STD-positive cases: All participants with positive tests for GC, CT, MG will be contacted by research staff, notified of their positive test result and referred to their physician in the clinic for an examination and treatment. By law, the Johns Hopkins International STD Research lab and the research team have an obligation to report positive cases of GC/CT to the Baltimore City Health Department (BCHD). Faxed reports will be sent to the health department.

5. Partner Notification and Treatment: All participants will be encouraged to notify their partners for treatment at baseline as a part of standard of care. The TECH-N will provide additional support and resources for notification and treatment if indicated. We will obtain self-reported partner notification and treatment data from each participant at the 14-day research visit.

6. Specimen Storage: According to the JHU PID protocol, patients should have a CBC, CRP, RPR, and HIV testing at the time of PID diagnosis. Three additional tube of blood (~10cc) will be drawn at the time of specimen collection and/or at the time of enrollment for storage and future RNA analysis and antibody testing.

Sera from the TECH-N study will be analyzed using established CDC methods to determine whether antibody detection to pgp-3 correlates with active CT infection in PID patients. Sera will be incubated with chemically modified microspheres (Luminex Corp., Austin, TX) conjugated to the Ct antigens pgp3. After washing out unbound serum antibodies, bound antibody will be detected with biotinylated mouse anti-human IgG (clone H2; Southern Biotech, Birmingham, AL) and biotinylated mouse anti-human IgG_4 (clone HP6025; Invitrogen, South San Francisco, CA), followed by *R*-phycoerythrin-labeled streptavidin (SAPE, Invitrogen, South San Francisco, CA). Beads are suspended in 125 µl PBS, shaken, and immediately read on a BioPlex 200 instrument (Bio-Rad, Hercules, CA) equipped with Bio-Plex Manager 6.0 software (Bio-Rad). Cut off ratios will be determined by positive and negative controls. If lateral flow POC cartridges are made available by researchers at CDC (anticipated), sera will be tested on this platform as well.

b. Study duration and number of study visits required of research participants. Each participant will be followed for 90 days. After enrollment, girls in the intervention arm will have 3 study visits (1 by the community health nurse within 5 days of enrollment/diagnosis of PID and 3 research visits at 2 weeks, 30 days, and 3 months and those in the control condition will have 3 research visits at 2 weeks and 3 months. Girls in the control condition will be advised to arrange their own follow-up care with their primary care provider according to current standards of care.

c. Blinding, including justification for blinding or not blinding the trial, if applicable. All data will be managed by an institutional data management service. While the principal investigator is blinded to individual study assignment; per protocol if a patient has a clinical problem while the CHN is in the field individual assignment will be unblended so that Drs. Butz and/or Trent will can assist with patient management.
d. Justification of why participants will not receive routine care or will have current therapy stopped.
e. Justification for inclusion of a placebo or non-treatment group. A control group is required to determine the efficacy of the intervention, however, in the control condition participants will receive optimized standard of care according to national and institutional guidelines. Definition of treatment failure or participant removal criteria. We will use an intention to treat approach so that all patients who enrolled will be followed until the end as our ability to maintain communication with them represents a key adherence behavior. Participants who indicate that they no longer wish to participate in the trial will be the only individuals that would be removed from the study.
f. Description of what happens to participants receiving therapy when study ends or if a participant's participation in the study ends prematurely. After the final study visit, participants will resume usual clinical care. If a participant ends the study prematurely during the standard treatment period for PID care they will be encouraged to follow-up with their primary care provider according to national standards (Centers for Disease Control and Prevention). Individuals who prematurely end their participation after the first 2 weeks; will be encouraged to follow-up with their primary care provider for follow-up clinical care per national guidelines (3-4 months after PID diagnosis) for routine STI re-screening and care.

7. *Mycoplasma* genitalium **macrolide resistance testing [Validation/Reliability Sub-study]**

De-identified waste samples testing positive for *Mycoplasma* **genitalium in the main study** will be screened for potential resistance to macrolides (Azithromycin) utilizing a novel high-resolution melting curve analysis (HRMA) assay that inerogates a region of the 23S rRNA gene known to contain point mutations conferring macrolide resistance to *M.* **genitalium** using the following protocol in the Johns Hopkins International STI and Biothreat Laboratory.

DNA from positive *M.* **genitalium** samples will be extracted via the Roche MagNA Pure LC robot utilizing the DNA I isolation kit following the manufacturer instructions with 200 µl of sample

input and 100 µl of elution volume. Lightscanner PCR for melting curve analysis: Primers for melting curve analysis are as follows: Mg23SF 5'CGGTGAAATCCAGGTACGG3' and MG23SR 5' CAGTAAAGCTTCACGGGGTCT3', with the following sequence as the predicted PCR product: 5'CGGTGAAATCCAGGTACGGGTGAAGACACCCGTTAGGC**GC**AACGGGACGGAAAGACCCCGTGAAGCTTTACTG3', with the base pairs responsible for macrolide resistance highlighted in red. The primers and predicated PCR product is modified from J Clin Microbiol 2014, 52 (5), 1549-1555. Lighscanner reactions will be prepared using the following: 4 µl of 2.5X Lighscanner Mix, 1 ul of each primer at 1.5 µM concentration, 1 µl of Lightscanner grade water, 1 µl of temperature calibrators at 1.5 µM and 2 µl of template DNA. Reaction conditions will consist of: 95°C for 5 minutes followed by 45 cycles of 95°C for 10 seconds, 58°C for 30 seconds and 72°C for 1 minute, followed by a 4°C cooling step for plate handling. HRMA analysis will be performed on Lighscanner plates using the Lightscanner (Biofire Diagnostics) per manufacturer instructions for small amplicons. Macrolide resistance will be determined by shifts in melting temperatures of generated PCR products compared to control reactions containing both wild type, and mutant 23S rRNA sequences for *M.* **genitalium**.

Since the results from resistance testing will not be available for patient and/or provider use as these methods have not been validated and/or approved for use by the FDA.

5. Inclusion/Exclusion Criteria

Inclusion Criteria: Eligible participants should be aged 13-21 diagnosed with mild-moderate PID, given an outpatient treatment disposition, permanently reside in the Baltimore Metropolitan area, and be willing to sign informed consent (including communication with the adolescent's primary care provider). Informed consent includes being willing to complete study procedures; including randomization and community-based follow-up by our team.

Exclusion Criteria: Patients who are pregnant, have a concurrent diagnosis of sexual assault, or are unable to communicate

6. Drugs/ Substances/ Devices

a. The rationale for choosing the drug and dose or for choosing the device to be used. **N/A**
b. Justification and safety information if FDA approved drugs will be administered for non-FDA approved indications or if doses or routes of administration or participant populations are changed. **N/A**
c. Justification and safety information if non-FDA approved drugs without an IND will be administered. **N/A**

7. Study Statistics

a. Primary outcome variable. **Adherence to 3-5 day visit; STI infection rate at 3 months**
b. Secondary outcome variables. **Cost differentials between study groups**
c. Statistical plan including sample size justification and interim data analysis.

Sample Size and Power: We conducted power analyses for Specific Aim 1, hypothesis 2. As described in the Preliminary Studies, we conducted a longitudinal analysis of data to examine repeat STIs/PID following PID diagnosed in pediatric ambulatory settings in Baltimore. The results suggested that at 3 months, the STI positivity rate was 25% and thus we will assume a 25% STI infection rate for the control group. Our preliminary data also suggests that at baseline we will be able to recruit approximately 175 participants in each arm for a total of 350 participants. We expect about 30% attrition over the study period and thus each study arm will have an effective sample size of 122.5 participants, for a total of 245 participants. Using these assumptions, our power calculations suggest that we will have 80% power to detect a relative risk of 1.66.

Data Analysis Primary Aim:

Overview: The overall approach will be an intention-to-treat analysis. To test the hypotheses, in addition to data cleaning and exploratory data analysis we will first need to address the challenges presented by the study design. The challenges include assessing the "success" of the randomization scheme, testing for differences in basic demographic characteristics between the clinical sites, and exploring missing data due to survey non-response and due to attrition. The plan for addressing each of the challenges and for hypothesis testing is described below. Data analysis for hypotheses 1 and 2 will be performed using STATA or comparable statistical software and analysis for hypothesis 3 will be performed using Tree Age software.

Testing for Differences by Randomization Assignment and Clinical Site: Comparability of the randomization assignments will be statistically assessed. If the randomization assignment does not result in equivalent groups or if there are significant differences by clinical site, we will adjust for nonequivalence in hypothesis testing (see below).

Missing Data due to Survey Non-response: The extent of data missing due to survey non-response will be assessed and if extensive, we will explore using imputation to deal with the missing data. We will take care to enter into imputation procedures with an exploratory yet principled approach as a naïve or unprincipled imputation method may create more problems than it solves distorting estimates standard errors and hypothesis tests. We will explore the use of Rubin's multiple imputation procedure, as one example.

Attrition Analysis: We will examine the extent of attrition over the course of the study. In particular, we will examine whether missing values are not missing at random and are non-ignorable. Since the study participants who drop out from the study may be different from those who remain, the propensity score method will be used to reduce selection bias, balancing the covariates in the data analysis.

Hypothesis testing:

In Aim 1, Hypothesis 1, we will assess whether adolescents with PID in the TECH-N intervention show greater adherence, defined as adherence to the 3 -5 day follow-up visit, compared to adolescents with PID in the control group. We will conduct a logistic regression analysis to determine whether the intervention group showed greater short-term adherence rate compared to the control group including potential factors that may affect the difference in rates. In Hypothesis 2, we will determine whether adolescent women with PID provided with the TECH-N intervention have a lower rate of recurrent STIs at 90 days compared to adolescents in

the control group. We will conduct a logistic regression analysis to determine whether the intervention group showed a lower rate of recurrent STIs at 90 days compared to the control group, including potential factors that may affect the difference in rates. In an exploratory analysis, we will also determine whether the adolescent women with PID in the TECH-N group have on average over time – baseline, 30-day, and 90-day- lower rates of recurrent STIs as compared to the intervention group. This initial analysis will use logistic regression and generalized estimating equations to handle the correlation of multiple measures within a person over time. We will also explore the group – intervention vs. control – trajectories over time to determine whether the two groups have statistically different slopes of recurrent STIs. We will also explore stratifying the curves by measures such as race/ethnicity and level of poverty. In Aim 2, Hypothesis 3, we will determine whether the TECH-N intervention is more cost-effective than the control for the prevention of recurrent STIs and PID-related complications. The cost evaluation will examine the net difference in costs between TECH-N and control groups in relation to prevention of 1) recurrent STIs (including PID) and 2) PID-related complications (e.g. re-hospitalization, medical visits, ectopic pregnancies). The difference in costs will account for the net difference between the outpatient strategies in use of medical services and indirect costs for PID treatment. The analysis will also consider savings in medical-care costs and indirect costs from prevention of the outcome variables. Two types of outcomes can be created. While the workplace and home productivity improvements are not the only monetary improvements associated with better care, a cost-benefit analysis can be performed simply comparing the excess costs of TECH-N with the medical care savings and productivity gains from cases averted. The distribution around the point estimate of the net benefits can be ascertained using a bootstrapping approach to account for the lack of the normal distribution that is likely in a net benefit calculation. We can also extend the analysis to a cost-effectiveness evaluation if TECH-N remains more expensive even after accounting for medical care costs and productivity loss averted. In this case, we can measure the dollars spent per case of STI averted over the relevant time period. We can also use published parameters to determine how the TECH-N intervention compares to hospitalization. Decision makers can then focus their attention on whether the added costs appear to be worth the health improvement. In this case, we could also use a bootstrapping analysis to calculate the distribution of the ICER.

Analyses will be performed to correlate positive antibody for pgp-3 with active CT infection at baseline for females in the TECH-N study.

a. Early stopping rules. A data safety monitoring board consisting of senior researchers in related fields and who have no direct involvement with the study will review study data including preliminary outcomes and adverse events. While all enrolled girls will be followed until the end of the follow-up period, preliminary analyses that demonstrate significant adverse findings prior to reaching sample size the team will discuss discontinuation of the trial. However, given the multiple pre-determined hypotheses related to cost-effectiveness, we will need to complete data collection even if we determine that significant findings for the primary outcomes (adherence/STI reduction).

8. Risks

a. Medical risks, listing all procedures, their major and minor risks and expected frequency. There are no medical risks to participation in this study. Collection of self-collected vaginal specimens, as a part of routine care, may produce some discomfort during specimen collection, particularly if infected. While we are examining STI infection as our outcome, girls in the trial are being tested according to the recommendations for re-screening and the risk is no greater than if done in traditional clinical care and/or public health settings. There is potential for breeches of confidentiality, but miniscule given the limited number of personnel with access to participant information. While the study will attempt to preserve patient confidentiality, this

study involves community based and/or home visits for most adolescents therefore disclosure of study participation is encouraged. In the pilot study for this work and the our published RCT of over 150 adolescents, we followed adolescent girls with PID in their home by either nursing staff or disease intervention specialists as a part of our public health approach to PID using structured outreach protocols without complaints of undue exposure, confidentiality breach, or complaints by parents/guardians about their adolescent's participation in the study.

b. Steps taken to minimize the risks.

All participants will provide informed consent and will be informed that their participation in the study will have no effect on subsequent medical care at any referral site. All patient data will be kept confidential and secure. A expert advisory panel will evaluate the progress of interventional trial(s), including periodic assessments of data quality and timeliness, participant recruitment, accrual and retention, participant risk versus benefit, performance of trial sites, and other factors that can affect study outcome. The research team on this project has been educated in the protection of human research participants and the treatment of protected health information as evidence by completion of their institutional compliance courses. New research staff will complete the Johns Hopkins Committee on Clinical Investigation courses as a part of their training and be added to the protocol for review accordingly.

Maryland state law permits an adolescent under 18 years old to consent herself for research when receiving reproductive care and most adolescents being seen in ambulatory sites at Johns Hopkins seek care unaccompanied by a parent, we will not require parental consent for participation in this study. The Johns Hopkins Institutional Review Board approved this approach for the successful pilot of this study design. Due to the longitudinal approach involved in this study, we encourage adolescents to keep parents informed about their participation in the study. As with the pilot study, we will explain to the adolescents that while they can consent to participate in the study, their parent would need to approve a visit by a health professional within their home. When the nurse interventionist contacts the adolescent to schedule the appointment, she will offer to explain to the parent that her daughter agreed to participate in a study through the Johns Hopkins University and that we would like to come to the home to complete the study visit. Oral consent via phone or in-person would be equivalent. If an adolescent wants to participate but expresses any concerns about home visits at enrollment or during the follow-up call with the nurse interventionist, the option will be made for visits to occur at an alternate location. As a part of our previous work, patients could meet the patient at the clinic, a relative's home, and/or a Baltimore City Health Department school health clinic.

Collection of self-collected samples is a routine part of clinical care. There are no additional steps, which can be taken to reduce discomfort associated with provision of a sample. Only members of our evaluation team will have access to the patient chart and data extraction forms. Involved personnel will be trained and committed to confidentiality and protection of patient rights and have received formal training through the institution. No identifying patient data will be included in any of the presentations or publications that may be generated as a consequence of this study. Our database will not contain any patient identifiers, as all participants will be assigned unique identifiers. Further, the study design provides for several alternatives for study participation that allow the adolescent to make decisions about non-participation, participation with parental awareness/involvement, and participation through alternate community outreach strategies. By offering these alternative strategies, our team is ensuring protection of privacy as afforded by law while setting the stage for adult involvement that could potentially facilitate improved clinical outcomes for the patient. We will document the strategy to be employed by

the team at the time of recruitment and evaluate potential differential impacts observed based on follow-up approach. Even if adolescents randomized to the TECH-N intervention arm choose not to have the visit done at home, they will still have their 3-5 day follow-up conducted by the TECH-N nurse and access to him/her during the follow-up period as all other participants in this arm. We will uses SMS services from HealthCloudSMS, a ZeroSum Health, LLC product, provides a reliable, secure, and cost-effective solution for researchers to implement text message based interventions. ZeroSum Health Security Policies meet or exceed HIPAA's privacy and security standards. The research team will access HealthCloudSMS through a secure web portal, where we can track participant progress. All data is encrypted before transmission, protected with SSL encryption during transmission, and is stored with 256-bit AES encryption on a dedicated, secure data server. The web application is hosted on a separate, secure application server. Both servers can only be accessed using key-based authentication (a 2048 bit RSA key pair), which facilitates highly secure access to ensure that PHI and all data is protected. HealthCloudSMS employs highly restrictive network access, a rigorous data backup protocol, and strong auditing capabilities, including detailed activity logs of database and application access. Finally, all participants will be encouraged to use standard safety mechanisms such as a pin or password to lock access to their cell phones by others.

c. Plan for reporting unanticipated problems or study deviations.
Any unanticipated problems or adverse events will be reported to the IRB immediately. Deviations from the original study protocol will be requested through a change in research request.

d. Legal risks such as the risks that would be associated with breach of confidentiality.

There are no anticipated legal risks associated with study participation. We have been following girls in the community for many years and in their homes without consequence as we have put safeguards in place to protect confidentiality as outlined above.

e. Financial risks to the participants. There are no anticipated financial risks to participations that are unaccounted for in the support and remuneration plan.

9. Benefits

a. Description of the probable benefits for the participant and for society.

The proposed research is the first to measure, in a randomized clinical trial, the effectiveness of a technology enhanced community nursing strategy for short-term clinical management of PID in adolescents. If successful, this study will yield data to support adoption of an alternative cost conscious, but effective strategy for managing adolescents with a complicated STI in the outpatient setting in accordance with CDC recommendations. To date, the data clearly demonstrates that adolescents need additional supports to self-manage PID in the outpatient setting. While adolescents engage in technology use, our preliminary work suggests that this is more likely when there is concurrent face-to-face interaction and/or a relationship that establishes a context for communication. This intervention provides a structure for the delivery of the necessary follow-up care, overcomes many of the barriers (e.g. lack of medical home, transportation) that often undermine adolescents ability to adhere, and provides for protection of confidentiality. The study also explores untested theories about acceptability of adult involvement after diagnosis of PID and the use of technology to communicate with a health provider during an acute medical illness in a community where the reproductive life course for young women is disproportionately influenced by

STI histories and for whom the cost-effectiveness thresholds for cost-effectiveness of a new intervention have not yet been determined.

Antibody testing may result in a viable CT point of care test in the future which will optimize clinical care in real time and prevent PID in general population samples.

10. Payment and Remuneration

Patients will receive a $10 gift card at enrollment and $10 for completed face-to-face research visit (14, 30,and 90 days) plus an addition $10 for each STI sample (N=3)provided according to the schedule as outlined below. Adolescents in the TECH-N arm will not receive remuneration for the 3-5-day clinical care visit given the current standards of care and because adherence to the CHN clinical visit is a behavior under study that must be made without any form of additional incentive. Patients will be remunerated $10 for each completed and/or in-person quarterly interview after the 3 month research visit. Participants who complete all 4 interviews for the first year will receive an added bonus of $50.

Adolescents who are eligible for the trial and assigned to the intervention arm, but do not have a cell phone will be provided with a disposable cell phone during the 1 month during which messages are sent by CFL. We anticipate that this will be a small number of adolescents given that CTIA asserts that there is 93% cell phone penetration in the United States 56 and 88% of the girls approached for our Depo-Text study at JHU had a cell phone for their personal use. 96 We have found, however, that maintenance of service can be a problem for low-income youth. We will also encourage enrolled participants to notify us if their cell phone service is discontinued during the intervention month so that we can ensure the delivery of CFL messages through use of either a cell phone renewal card and/or use of a disposable phone. The rates of remuneration for interviews and specimen collection are based on our previous field experience with adolescents and young adults in Baltimore who often have competing agendas (e.g. part-time employment) that may preclude them from participating in study visits.

11. Costs

a. Detail costs of study procedure(s) or drug (s) or substance(s) to participants and identify who will pay for them.

There are no anticipated costs to participants that are unaccounted for in the support and remuneration plan for the study and/or usual care.
