# Supplementary material for: Efficacy of a Technology-Enhanced Community Health Nursing Intervention vs Standard of Care for Female Adolescents and Young Adults With Pelvic Inflammatory Disease: A Randomized Clinical Trial
Source: JAMA Netw Open. 2019 Aug 7;2(8):e198652. doi: 10.1001/jamanetworkopen.2019.8652 (PMC6686980; doi:10.1001/jamanetworkopen.2019.8652)
Supplement: Supplement 2. — eTable 1. Sample Automated Text Support Messages Sent to 286 Women With Pelvic Inflammatory Diseases Enrolled in the Technology-Enhanced Community Health Nursing (TECH- N) Trial eTable 2. Predicted Change From Baseline STI Positivity at 30- and 90-Day Follow-up Among 286 Women With Pelvic Inflammatory Diseases Enrolled in the Technology-Enhanced Community Health Nursing (TECH- N) Trial eTable 3. Baseline and 3-Month Condom Use at Last Sexual Intercourse, by Intervention Group and for All Study Participants [file jamanetwopen-2-e198652-s002.pdf]

Supplementary Online Content

Trent M, Perin J, Gaydos CA, et al. Efficacy of a technology-enhanced community health nursing intervention vs standard of care for female adolescents and young adults with pelvic inflammatory disease: a randomized clinical trial. *JAMA Netw Open.* 2019;2(8):e198652. doi:10.1001/jamanetworkopen.2019.8652

eTable 1. Sample Automated Text Support Messages Sent to 286 Women With Pelvic Inflammatory Diseases Enrolled in the Technology-Enhanced Community Health Nursing (TECH- N) Trial

eTable 2. Predicted Change From Baseline STI Positivity at 30- and 90-Day Follow-up Among 286 Women With Pelvic Inflammatory Diseases Enrolled in the Technology-Enhanced Community Health Nursing (TECH- N) Trial

eTable 3. Baseline and 3-Month Condom Use at Last Sexual Intercourse, by Intervention Group and for All Study Participants

This supplementary material has been provided by the authors to give readers additional information about their work.

© 2019 Trent M et al. *JAMA Network Open*.

eTable 1. Sample Automated Text Support Messages Sent to 286 Women With Pelvic Inflammatory Diseases Enrolled in the Technology-Enhanced Community Health Nursing (TECH- N) Trial
STDs = Sexually Transmitted Diseases.

Welcome Message	Thank you for enrolling in the TECH-N study. We will contact you within 24 hours to arrange a follow-up visit. Call us at --- if you don't receive a call. Text 1 if you got this message. TECH-N Team.
Daily Adherence Reminder	Good morning! Don't forget to take your medication twice today with a BIG glass of water. TECH-N Nurses
Daily Dosage Inquiry	Good evening! How many doses did you take today? Text 0, 1, or 2. TECH-N Nurses.
Intervention Message 1 (Taking all Medications)	That's great! You're on your way to recovering completely.
Intervention Message 2 (Taking only 1 dose of Medication)	One dose is good, but you need to take both doses to make sure your body heals properly.
Sexual Health Message 1	Condoms prevent STDs. Stop by the TECH-N Office if you need some. Call XXX-XXX-XXXX to let us know are coming by. TECH-N Team
Sexual Health Message 2	Birth control is a healthy part of a relationship. Call the Title 10 Clinic at XXX-XXX-XXXX if you need family planning help. TECH-N Team

© 2019 Trent M et al. *JAMA Network Open*.

eTable 2. Predicted Change From Baseline STI Positivity at 30- and 90-Day Follow-up Among 286 Women With Pelvic Inflammatory Diseases Enrolled in the Technology-Enhanced Community Health Nursing (TECH- N) Trial

	30-day follow-up		90-day follow-up		
	**Intervention**	**Control**	**Intervention**	**Control**	***p****
**Predicted change in prevalence from baseline**					
**Chlamydia****	-17.4% (-25 ,-10)	-7.1% (-13 ,-2)	-27.6% (-36 ,-20)	-13.7% (-21 ,-6)	0.145
**Gonorrhea****	-3.3% (-6 ,-0)	-1.2% (-4 ,1)	-6.5% (-11 ,-2)	-3.2% (-9 ,3)	0.155
**Chlamydia or Gonorrhea**	-15.8% (-22 ,-9)	-5.4% (-10 ,-1)	-28.3% (-36 ,-20)	-12.7% (-21 ,-4)	0.021

* Test for difference between arms in average trend over the study period, using generalized estimating equations to account for variability within individuals.

© 2019 Trent M et al. *JAMA Network Open*.

eTable 3. Baseline and 3-Month Condom Use at Last Sexual Intercourse, by Intervention Group and for All Study Participants

Reported among 286 women with pelvic inflammatory diseases enrolled in the technology-enhanced community health nursing (TECH- N) trial

	**Overall**		**Intervention**		**Control**		
	**Total**	**N (%)**	**Total**	**N (%)**	**Total**	**N (%)**	***p****
**Condom use at Baseline†**	279	48 (17%)	146	23 (16%)	133	25 (19%)	0.607
**Condom use at 3 months††**	259	86 (33%)	134	49 (37%)	125	37 (30%)	0.290
**Change in condom use from baseline to 3 months****		16%		21%		11%	0.100

* Significance determined using Chi-square test.
** Significance determined by generalized estimating equations.
† Number of observations vary due to participant non-response.
†† Number of observations vary due to loss to follow-up of 24 (8% of 286) women, and to participant non-response

© 2019 Trent M et al. *JAMA Network Open*.
