# Supplementary material for: Efficacy of a Technology-Enhanced Community Health Nursing Intervention vs Standard of Care for Female Adolescents and Young Adults With Pelvic Inflammatory Disease: A Randomized Clinical Trial
Source: JAMA Netw Open. 2019 Aug 7;2(8):e198652. doi: 10.1001/jamanetworkopen.2019.8652 (PMC6686980; doi:10.1001/jamanetworkopen.2019.8652)
Supplement: Supplement 3. — Data Sharing Statement [file jamanetwopen-2-e198652-s003.pdf]

Data Sharing Statement

Data

Data available: Yes
Data types: Deidentified participant data, Data dictionary
How to access data: Individual requests for data will be considered on a case-by-case basis as appropriate to the research protocol. All requested data will be provided with appropriate documentation to prevent misuse, misinterpretation, and/ or confusion. All project data will be retained for a minimum of five years after project completion for data analysis by the primary research team.
When available: beginning date: 11-30-2023

Supporting Documents

Document types: None

Additional Information

Who can access the data: Researchers whose proposed use of the data has been approved.
Types of analyses: Non-commercial health-related research.
Mechanisms of data availability: With investigator support, approval of proposal, and signed data access agreement.
Any additional restrictions: N/A
